# Investigating the factors that explain white matter hyperintensity load in older Indians

**DOI:** 10.1093/braincomms/fcad008

**Published:** 2023-01-18

**Authors:** Leon Aksman, Kirsten Lynch, Arthur Toga, Aparajit Ballav Dey, Jinkook Lee

**Affiliations:** Mark and Mary Stevens Neuroimaging and Informatics Institute, Keck School of Medicine, University of Southern California, Los Angeles, CA 90033, USA; Mark and Mary Stevens Neuroimaging and Informatics Institute, Keck School of Medicine, University of Southern California, Los Angeles, CA 90033, USA; Mark and Mary Stevens Neuroimaging and Informatics Institute, Keck School of Medicine, University of Southern California, Los Angeles, CA 90033, USA; Geriatric Medicine, All India Institute of Medical Sciences, New Delhi, Delhi 110029, India; Dana and David Dornsife College of Letters, Arts and Sciences, University of Southern California, Los Angeles, CA 90089, USA

**Keywords:** dementia risk factors, MRI, white matter hyperintensities, cerebrovascular pathology, low- and middle-income countries

## Abstract

White matter hyperintensities are areas of hyperintense signal on MRI that typically represent cerebrovascular pathology. While focal white matter hyperintensities are common among older individuals, extensive white matter hyperintensities have been found to accelerate the progression of dementia. However, little is currently known about how various socioeconomic, health, lifestyle and environmental factors affect the severity of these lesions, particularly in low- and middle-income countries such as India. We investigated this question using cross-sectional MRI data (*n* = 126) from a pilot neuroimaging sub-study of an ongoing, nationally representative epidemiological study of late-life cognition in India. As a screening step, we estimated white matter hyperintensity load from fluid-attenuated inversion recovery MRI using a fully automated technique and tested for associations with each factor separately, controlling for age, sex and estimated total intracranial volume in each case. A combined model of white matter hyperintensity load included five factors which were significant after multiple comparisons correction: systolic blood pressure, body mass index, urbanicity status (urban versus rural living), daily chore hours and the frequency of store trips. This model explained an additional 27% of the variance in white matter hyperintensity load (54 versus 27% for the baseline model with only age, sex and estimated total intracranial volume). We accounted for the possibility of reverse causality by additionally controlling for concurrent markers of neurodegeneration and cognitive impairment, with no substantial change in our findings. Overall, our findings suggest that controlling high blood pressure and maintaining both a healthy body mass index and high levels of physical activity may reduce white matter hyperintensity load in older Indian adults, helping to prevent or delay dementia.

## Teaser

Aksman *et al.* investigated which socioeconomic, health and lifestyle factors affect the white matter hyperintensity (WMH) load in older Indians, finding that high blood pressure, high body mass index and low levels of physical activity contribute to WMH load in this population.

## Graphical Abstract

**Graphical abstract:**
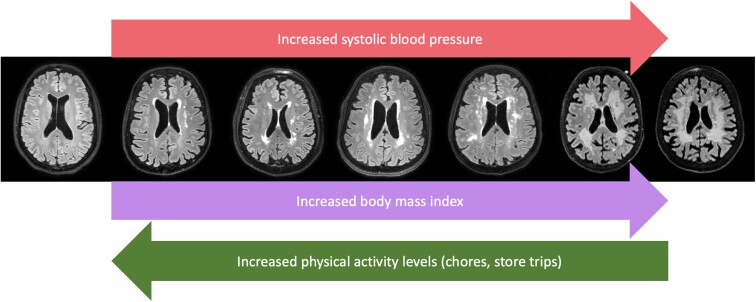

## Introduction

White matter hyperintensities (WMHs) are areas of the hyperintense signal that are visible on T_2_-weighted (T2w), T_2_* proton density and fluid-attenuated inversion recovery (FLAIR) sequence MRI.^1^ While small, focal WMHs are common among older individuals,^2,3^ extensive WMHs typically reflect cerebrovascular pathology due to cerebral small vessel disease.^4–6^ Cerebrovascular pathologies have been recognized as important comorbidities that lower the threshold for developing dementia.^7^ While hypertension has consistently been identified as the most important modifiable risk factor for cerebrovascular pathology,^8^ older adults with normal blood pressure (BP) may also present with severe WMHs. Overall, BP and related vascular risk factors appear to explain only a small percentage of the variance of WMH load.^9^ It is, therefore, essential to build a more complete picture of the factors that may influence severity of these lesions. This is particularly true in low- and middle-income countries (LMIC) such as India, which have been under-represented in neuroscientific studies.^10^

Studies linking histopathology with post-mortem MRI have found that WMHs reflect a wide variety of underlying pathologies. These include demyelination, loss of axons and oligodendrocytes, dilated perivascular spaces and glial cell response.^11^ The proposed pathogenic mechanisms for WMHs include vascular dysfunction, inflammation and blood–brain barrier dysfunction; these mechanisms have also been implicated in dementia.^12^ Indeed, neuropathologic studies show that cerebrovascular pathology is present in the majority of dementia patients.^13^ However, it is unclear whether WMHs independently affect cognitive decline or whether they interact with Alzheimer's disease (AD) pathologies such as amyloid-β to worsen or decline.^14,12^ WMHs may cause dementia by disrupting white matter tracts;^15^ alternatively, parietal WMHs in AD patients may result from neurofibrillary tangles and neuronal loss that causes axonal loss.^16^

In this study, we set out to identify a broader set of risk βfactors influencing WMH load. Our study was motivated by the 2020 Lancet Commission report on dementia, which identified 12 modifiable risk factors that account for roughly 40% of worldwide dementia.^17^ These were low education in early life; hearing loss, traumatic brain injury, hypertension, excessive alcohol consumption and obesity in mid-life; and smoking, depression, social isolation, physical inactivity, diabetes and air pollution in later life. We hypothesized that many of these factors may be associated with cerebrovascular pathology, which may in turn affect dementia.

Using pilot neuroimaging data from a subset of an ongoing epidemiological study of ageing and dementia in India, we associated WMH load with a diverse set of measures, covering 11 of the 12 factors in the Lancet Commission report (excluding only traumatic brain injury). These included measures of socioeconomic status, health, lifestyle and environmental exposure to air pollution. India provides an excellent setting to understand these factors, given the high prevalence of hypertension, diverse socioeconomic conditions (in terms of both urbanicity and literacy) and high levels of air pollution in cities. Our goals were to (i) understand the relative importance of each factor by comparing how much additional variance in WMH load each factor explained and (ii) develop a single model of WMH load using the most explanatory factors from the first step. We found that systolic BP, body mass index (BMI), physical activity levels and urbanicity all helped to explain the volume of WMHs.

## Materials and methods

We analysed data from the Harmonized Diagnostic Assessment of Dementia for the Longitudinal Aging Study in India (LASI-DAD), a nationally representative and publicly available data set on late-life cognition and dementia in India.^18^ LASI-DAD, a sub-study of the main LASI study (*n* = 70 000 participants aged 45+), has drawn a sample of 4096 community-residing older adults from 19 states in India, representing 91.6% of the ethnically diverse population. The inclusion criteria were age 60+, with no exclusion criteria. LASI-DAD has administered the Harmonized Cognitive Assessment Protocol, a common cognitive test battery used by an international network of researchers.^19^ LASI-DAD participants were classified as high or low risk of cognitive impairment based on cognitive tests from the main LASI study or proxy report for those who did not complete cognitive testing, as described by Lee *et al*.^20^ LASI-DAD has collected data on a variety of modifiable risk factors of dementia, including geriatric clinical assessment, obesity and hypertension, questions on functional health such as basic and instrumental activities of daily living, physical activity levels, hearing, literacy, depression, anxiety, diet and exposure to indoor- and outdoor air pollution.

LASI-DAD is one of the first population-based studies to collect neuroimaging data for a sub-sample of participants (*n* = 137 in total). Urban and rural participants were recruited from within a 4 h driving distance of three urban scanning sites located in Bangalore (National Institute of Mental Health and Neuroscience), Mumbai (NM Medical Center) and Kolkata (Institute of Neuroscience). The study used the Alzheimer’s disease neuroimaging initiative-3 protocol for the Phillips Ingenia 3 T and Siemens Skyra 3 T scanners with 32-channel head coils.^20^ The 55 min protocol included T_1_-weighted, FLAIR, high-resolution 2D T_2_-weighted imaging of the hippocampus, resting-state functional, diffusion and susceptibility-weighted MRI. Here we report results from the FLAIR and T_1_-weighted MRI scans. FLAIR images were acquired with echo time (TE) = 221 ms; repitition time (TR) = 4800 ms; voxel size = 1 × 1 × 1 mm^2^; slice thickness = 1 mm; with GeneRalized Autocalibrating Partial Parallel Acquisition (GRAPPA) 2 acceleration. T_1_-weighted images were acquired in sagittal orientation using a three-dimensional magnetization prepared rapid acquisition gradient echo (MPRAGE) sequence (TE = 3.7 ms; TR = 8.0 ms; angle = 8°; voxel size = 1 × 1 × 1 mm^2^; field-of-view (FOV) = 24 cm; slice thickness = 1 mm; with GRAPPA 2 acceleration).

There were 129 sub-study participants with available FLAIR and T_1_-weighted MRI data. We estimated WMH volume using the lesion prediction algorithm (LPA),^21^ which is a fully automated algorithm that is part of the Lesion Segmentation Tool (www.statistical-modelling.de/lst.html) for statistical parametric mapping (SPM). The LPA required only a FLAIR image to estimate WMH volume. We excluded two images due to the low quality of image segmentation that could not be corrected with additional preprocessing steps and one image with corrupted header information. Thus, we had high-quality estimates of WMH volume for 126 participants, which were log-transformed to improve normality for statistical modelling. We refer to these log WMH volume estimates as WMH load. We used Freesurfer version 6 to segment the T1-weighted images to obtain the estimated bilateral hippocampal volume, cortical volume and estimated total intracranial volume (eTIV in mm^3^),^22,23^ which were used as covariates in our analyses.

We tested 25 socioeconomic, health, lifestyle and environmental measures that were measured in Wave 1 of the overall LASI-DAD study. We tested several demographic and physical health measures: urbanicity status (lives in an urban or rural setting), years of education, literacy status, systolic and diastolic BP, BMI and hearing test score (using a HearCheck device, sum of left ear and right ear scores). We tested several self-reported health and lifestyle measures: frequency of loneliness, depression, smoking and alcohol consumption. We also tested a number of health and lifestyle measures based on a computer-assisted personal interview of a family or friend whom the participant nominated as an informant. As roughly half (48%) of the LASI-DAD study’s participants are at high risk of cognitive impairment, the informant interview is considered to be a more reliable way to measure participants’ daily functions. These informant-reported measures were estimated time spent reading, watching television, performing chores (household chores, maintenance and gardening), using a computer, napping, exercising, walking, working or volunteering, going to the store or market, whether respondent prepares hot meals and whether they use public transportation. Finally, we tested three measures of indoor and outdoor air pollution: self-reported use of unclean cooking fuel (wood/coal) or unclean household fuel (kerosene, charcoal/lignite/coal, crop residue, wood/shrub or dung cake) and household level exposure to ambient air pollution (PM2.5 estimated from satellite and ground monitor data, measured in 2016). A full breakdown of all 25 measures is provided in Table 1.

**Table 1:** Participant characteristics of full LASI-DAD cohort and neuroimaging sub-study cohort

Variable	Units	Full (*N* = 4096)	Neuroimaging (*N* = 126)	Difference
Demographics				
Age	Years, mean (std) (min max)	69.9 (7.6) (60.0 105.0)	68.1 (6.0) (60.0 87.0)	*F* = 7.4, (0.007)
Sex	Female/male (%)	2207/1889 (54/46%)	58/68 (46/54%)	*χ* ^2^=3.0 (0.08)
Education years	Years, mean (std) (min max)	3.8 (4.7) (0.0 21.0)	5.5 (4.6) (0.0 17.0)	*F* = 15.2 (<0.001)
HMSE	Mean (std) (min max)	22.6 (5.5) (0.0 30.0)	25.4 (4.2) (10.0 30.0)	*F* = 32.3 (<0.001)
Risk of cognitive impairment	High/low (%)	1981/2115 (48/52%)	49/77 (39/61%)	*χ* ^2^ = 4.4 (0.04)
Literate	Yes/no (%)	1741/2355 (43/57%)	79/47 (63/37%)	*χ* ^2^= 20.3 (<0.001)
Urbanicity	Yes/no (%)	1557/2539 (38/62%)	93/33 (74/26%)	*χ* ^2^= 65.8 (<0.001)
Health measures				
Systolic BP	mmHg, mean (std) (min max)	140.5 (24.4) (75.0 232.0)	137.4 (20.2) (101.0 191.0)	*F* = 2.0 (0.16)
Diastolic BP	mmHg, mean (std) (min max)	81.7 (12.4) (43.0 155.0)	83.4 (12.1) (58.0 125.0)	*F* = 2.2 (0.14)
BMI	kg/m^2^, mean (std) (min max)	22.5 (5.1) (9.5 47.7)	24.0 (4.7) (13.4 37.4)	*F* = 10.8 (0.001)
Air pollution exposure (PM2.5)	μg/m^3^, mean (std) (min max)	72.0 (33.4) (26.0 153.1)	55.2 (14.2) (33.1 73.8)	*F* = 31.8 (<0.001)
Hearing test	Mean (std) (min max)	5.8 (2.4) (0.0 12.0)	5.8 (2.1) (1.0 12.0)	*F* = 0.1 (0.82)
Self-reported				
Weekly loneliness	Rarely/<1/1–2/3–4/5–7 days (%)	2397/898/411/316 (59/22/10/8%)	90/21/7/8 (71/17/6/6%)	*χ* ^2^= 6.8 (0.009)
Weekly depression	Rarely/<1/1–2/3–4/5–7 days (%)	1680/1395/590/359 (41/34/14/9%)	73/42/8/3 (58/33/6/2%)	*χ* ^2^= 18.8 (<0.001)
Smoking frequency	Cigarettes/day, mean (std) (min max)	1.6 (5.6) (0.0 144.0)	1.8 (4.8) (0.0 24.0)	*F* = 0.1 (0.72)
Alcohol frequency	Never/<1× per month/1–3 days per month/1–4 days per week/5 + days per week (%)	3793/84/72/59/54 (93/2/2/1/1%)	115/5/3/1/1 (91/4/2/1/1%)	*χ* ^2^= 0.3 (0.58)
Uses unclean cooking fuel	Yes/no (%)	1640/2456 (40/60%)	18/108 (14/86%)	*χ* ^2^= 34.0 (<0.001)
Uses unclean household fuel	Yes/no (%)	2333/1763 (57/43%)	45/81 (36/64%)	*χ* ^2^ = 22.4 (<0.001)
Informant-reported				
Exercise frequency	Never/rarely/1× a month/1× a week/>1× a week/daily (%)	3587/180/19/26/37/247 (88/4/0/1/1/6%)	107/9/1/0/0/9 (85/7/1/0/0/7%)	*χ* ^2^ = 0.7 (0.40)
Walking frequency	Never/rarely/1× a month/1× a week/>1× a week/daily (%)	2206/371/30/85/159/1245 (54/9/1/2/4/30%)	68/12/2/3/6/35 (54/10/2/2/5/28%)	*χ* ^2^ = 0.1 (0.80)
Work frequency	Never/rarely/1× a month/1× a week/>1× a week/daily (%)	2311/605/177/186/231/586 (56/15/4/5/6/14%)	47/19/3/7/15/35 (37/15/2/6/12/28%)	*χ* ^2^=25.2 (<0.001)
Store trip frequency	Never/rarely/1× a month/1× a week/>1× a week/daily (%)	1346/718/292/578/661/501 (33/18/7/14/16/12%)	20/18/11/15/39/23 (16/14/9/12/31/18%)	*χ* ^2^ = 25.1 (<0.001)
Daily TV hours	Never/0.5/1/2–3/4–6/7+ h (%)	1301/576/931/1016/211/61 (32/14/23/25/5/1%)	12/20/24/58/9/3 (10/16/19/46/7/2%)	*χ* ^2^ = 35.4 (<0.001)
Daily reading hours	Never/0.5/1/2–3/4–6/7+ h (%)	2841/512/529/177/31/6 (69/13/13/4/1/0%)	73/33/16/3/1/0 (58/26/13/2/1/0%)	*χ* ^2^ = 3.9 (0.05)
Daily chore hours	Never/0.5/1/2–3/4–6/7+ h (%)	1335/425/781/1013/371/171 (33/10/19/25/9/4%)	26/19/27/34/10/10 (21/15/21/27/8/8%)	*χ* ^2^ = 4.5 (0.03)
Daily computer hours	Never/0.5/1/2–3/4–6/7+ h (%)	3959/35/57/32/8/5 (97/1/1/1/0/0%)	121/3/0/2/0/0 (96/2/0/2/0/0%)	*χ* ^2^ = 0.1 (0.72)
Daily napping hours	Never/0.5/1/2–3/4–6/7+ h (%)	844/495/1185/980/312/280 (21/12/29/24/8/7%)	24/22/42/32/6/0 (19/17/33/25/5/0%)	*χ* ^2^ = 3.4 (0.06)
Prepares hot meals	Yes/no (%)	1643/2453 (40/60%)	50/76 (40/60%)	*χ* ^2^ = 0.0 (0.92)
Uses public transport	Yes/no (%)	2518/1578 (61/39%)	102/24 (81/19%)	*χ* ^2^ = 19.7 (<0.001)

For full cohort statistics: systolic BP *N* = 4017, diastolic BP *N* = 4004, BMI *N* = 3775, hearing *N* = 3916, smoking *N* = 4060, alcohol *N* = 4062, loneliness *N* = 4022, depression *N* = 4024. *N* = 4096 for all other full cohort variables. For neuroimaging cohort statistics: BMI *N* = 124, hearing *N* = 124, smoking *N* = 125, alcohol *N* = 125. *N* = 126 for all other neuroimaging cohort variables. Difference column contains relevant statistic (either *F* or *χ*^2^) and *P*-value for tests between full and neuroimaging cohort based on: (i) one-way ANOVA tests for: age, education years, HMSE, systolic BP, diastolic BP, BMI, air pollution, hearing and smoking; (ii) *χ*^2^ tests on: sex, risk of cognitive impairment, literacy, urbanicity, unclean cooking fuel, unclean household fuel, preparing hot meals and using public transport and (iii) Kruskal–Wallis tests on frequency of: alcohol use, loneliness, depression, exercise, walking, store trips, TV, reading, chores, computer use and napping.

### Statistical analysis

In order to understand how each of these measures affects WMH load (i.e. log WMH volume), we built a separate linear model for each factor with WMH load as the dependent variable and age, sex, head size (eTIV) and the measure in question as the independent variables. To compare measures in a meaningful way, we calculated each measure’s additional explained variance (*R*^2^) as the difference between its model’s explained variance and the baseline model’s explained variance. The baseline model contained age, sex and eTIV as independent variables. All reported *P*-values were corrected for multiple comparisons using MATLAB’s *mafdr* false discovery rate (FDR) function, setting the ‘Benjamini and Hochberg f alse d iscovery rate (BHFDR)’ flag to ‘true’ to enable Benjamini and Hochberg’s linear step-up procedure.^24^ Results were deemed significant at FDR *P*-value <0.05. To account for reverse causation, we repeated this analysis with a second baseline model which additionally controlled for markers of neurodegeneration (hippocampal volume, cortical volume) and cognitive impairment (HMSE)^25^.

We used the above procedure as a screening method for the final, combined model of WMH load. Before building the combined model, we computed the pairwise correlations between each of the factors that survived multiple comparisons correction. Because we were correlating different types of measures (continuous, categorical and binary), we used Spearman’s rank correlation in all cases. We then excluded several factors with very significant correlations to other factors in order to avoid collinearities in the combined model. We standardized both the dependent variable (WMH load) and the independent variables (age, sex, eTIV plus the identified factors) so that the coefficients of the combined model were standardized.

We investigated the differences between urban and rural participants within both the neuroimaging cohort and the whole LASI-DAD cohort, comparing the other 24 factors in both cases. To do this, we built a separate logistic regression for each factor with, in each case, urbanicity status (urban coded as one, rural as zero) as the dependent variable and age, sex and the given factor as the independent variables. We also investigated whether there was a selection bias in the recruitment of either urban or rural neuroimaging cohort participants relative to the overall LASI-DAD study. To do this, we compared the 24 factors between urban neuroimaging cohort participants (*N* = 93) and urban LASI-DAD participants who were not part of the neuroimaging study (*N* = 1463). We built a logistic regression model with a dependent variable that coded one for urban neuroimaging cohort participants and zero for all other urban participants (all rural participants were excluded from this analysis). Age, sex and the given factor were the independent variables in each case. Similarly, we tested for differences between rural neuroimaging cohort participants (*N* = 33) and rural LASI-DAD participants not in the neuroimaging cohort (*N* = 2506). In this case, the dependent variable was coding one for rural neuroimaging cohort participants and zero for all other rural participants (urban participants excluded). WMH load was not included in any of these three analyses.

## Results

Participant characteristics for both the neuroimaging cohort and the overall LASI-DAD cohort are shown in Table 1. The baseline model of WMH load (age, sex and eTIV) had an explained variance of 27% (Table 2). Increased age and eTIV were both associated with increased WMH load (age: *β* = 0.433, SE = 0.078, *P* < 0.001; eTIV: *β* = 0.377, SE = 0.097, *P* < 0.001).

**Table 2:** Baseline model of WMH load (*R*^2^ = 27%)

Variable	*β*	SE	*T*-statistic	*P*-value
Intercept	0.000	0.077	0.0	1.00
Age	0.433	0.078	5.5	<0.001
Sex	0.096	0.097	1.0	0.33
eTIV	0.377	0.097	3.9	<0.001

Table 3 shows associations between WMH load and 25 socioeconomic, health, lifestyle and environmental measures, each corrected for age, sex and eTIV. We found increased systolic and diastolic BP were both associated with increased WMH load, explaining an additional 10.7 and 5.8% of the variance, respectively (systolic BP: *t* = 4.6, FDR *P* < 0.001; diastolic *t* = 3.2, BP FDR *P* = 0.008). Similarly, increased BMI was associated with increased WMH load, explaining a 7.3% of the variance (*t* = 3.8, FDR *P* = 0.003). Increased daily physical activity in the form of performing household chores, maintenance or gardening was associated with reduced WMH load, explaining an additional 6.2% of the variance (*t* = −3.4, FDR *P* = 0.008). Living in an urban setting was also associated with increased WMH load, explaining an additional 5.9% of the variance (*t* = 3.2, FDR *P* = 0.03). Finally, we found negative associations between both the frequency of trips to the store or market (4.8% additional explained variance, *t* = −2.9, FDR *P* = 0.02) and the usage of public transportation (4.7% additional explained variance, *t* = −2.9, FDR *P* = 0.02). We found no significant associations with the other 18 factors after controlling for multiple comparisons.

**Table 3:** Associations between socioeconomic, health, lifestyle and environmental measures and WMH load based on a one-factor screening procedure

Variable	Additional *R*^2^ (%)	*T*-statistic	*P*-value	FDR *P*-value
Systolic BP	**10.7**	**4.6**	**<0.001**	**<0.001**
BMI	**7.3**	**3.8**	**<0.001**	**0.003**
Daily chore hours	**6.2**	**−3.4**	**0.001**	**0.008**
Urbanicity	**5.9**	**3.2**	**0.002**	**0.008**
Diastolic BP	**5.8**	**3.2**	**0.002**	**0.008**
Store trip frequency	**4.8**	**−2.9**	**0.004**	**0.02**
Uses public transport	**4.7**	**−2.9**	**0.005**	**0.02**
Work frequency	3.3	−2.4	0.02	0.05
Depressed frequency	3.2	2.4	0.02	0.05
Literate	2.3	2.0	0.05	0.13
Prepares hot meal	2.1	−1.9	0.06	0.14
Air pollution exposure (PM2.5)	1.9	1.8	0.07	0.15
Daily napping hours	1.1	1.3	0.19	0.36
Exercise frequency	0.6	1.0	0.33	0.60
Loneliness frequency	0.3	0.7	0.47	0.73
Uses unclean household fuel	0.3	−0.7	0.52	0.74
Daily TV hours	0.2	0.6	0.57	0.74
Daily reading hours	0.1	0.4	0.67	0.84
Walking frequency	0.1	0.3	0.76	0.91
Education years	0.0	0.0	1.00	1.00
Daily computer hours	0.0	−0.2	0.86	0.94
Uses unclean cooking fuel	0.0	−0.1	0.90	0.94
Smoking frequency	−0.4	−0.8	0.44	0.73
Hearing test	−0.6	−0.6	0.53	0.74
Alcohol frequency	−0.7	0.2	0.80	0.90

Each variable was used separately to predict WMH load, controlling for age, sex and eTIV in each case. Additional explained variance (*R*^2^) percentage is calculated as difference between resulting model’s *R*^2^ and baseline model’s (age, sex and eTIV) *R*^2^. Rows in bold are those that were significant after multiple comparisons correction, with FDR *P-*value <0.05.

**Figure 1:**
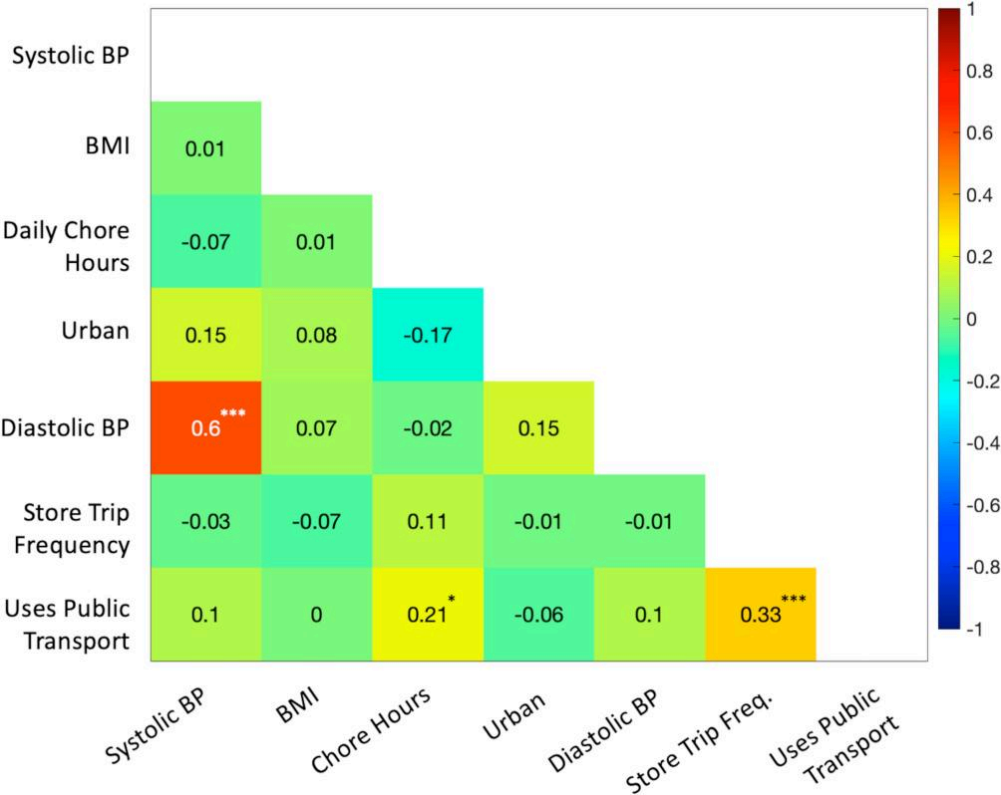
**Correlations between the seven explanatory factors of WMH load identified by the one-factor screening procedure.** Seven explanatory factors are from Table 3. Each cell in the figure depicts a pairwise Spearman’s rank correlation coefficient. For all cells: *P < 0.05, **P < 0.01 and ***P < 0.001.

**Figure 2:**
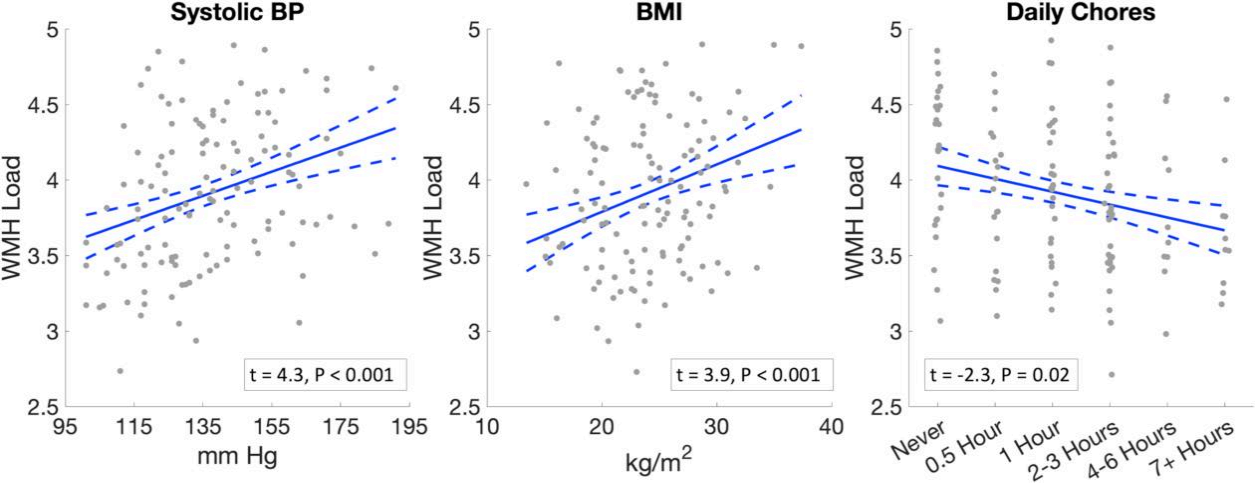
**Associations between WMH load and three of the most explanatory factors, based on the combined model in Table 4.** Daily chores’ categorical measures (*x*-axis) have been dithered about their true integer values for visualization. *T*-statistics and *P*-values depicted are from the combined linear regression model of Table 4.

**Figure 3:**
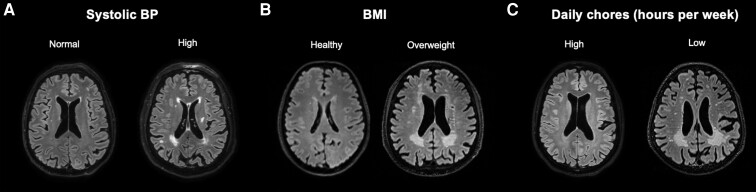
**Representative FLAIR axial slices in age- and sex-matched participants stratified by variables significantly associated with WMH load.** (**A**) Systolic BP: 66-year-old male with normal systolic BP of 119 mmHg (left); and 67-year-old male with elevated, clinically hypertensive systolic BP of 152 mmHg (right). (**B**) BMI: 63-year-old male with BMI of 19.6, falling within the healthy range (18.5–24.9); and 64-year-old male with BMI of 28.5 falling within the overweight range (25–29.9). (**C**) Sixty-two-year-old male with informant-reported >7 h of chores daily (left); and 63-year-old male with informant-reported no daily chore hours (right). Overall, we found increased WMH load in participants with elevated systolic BP, high BMI and fewer informant-reported daily chore hours.

Figure 1 depicts pairwise correlations between the seven factors we identified. As expected, systolic and diastolic BP were correlated (*ρ* = 0.60, *P* < 0.001) as were the frequency of store or market trips and the use of public transportation (*ρ* = 0.33, *P* < 0.001). We, therefore, excluded diastolic BP and public transportation use from the combined model to avoid collinearities. The combined model, presented in Table 4, included systolic BP, BMI, daily chore hours, urbanicity and store trip frequency in addition to age, sex and eTIV. This model explained an additional 27% of the variance in WMH load (54% for the combined model versus 27% for the baseline model). Within this model, each of the five factors was associated with WMH load (systolic BP: *β* = 0.278, SE = 0.064, *P* < 0.001; BMI: *β* = 0.262, SE = 0.068, *P* < 0.001; urbanicity: *β* = 0.169, SE = 0.068, *P* < 0.01; daily chore hours: *β* = −0.162, SE = 0.070, *P* = 0.02; store trip frequency: *β* = −0.154, SE = 0.067, *P* = 0.02). Figure 2 depicts the associations between WMH load and three of the most explanatory factors (systolic BP, BMI and daily chore hours) while Fig. 3 depicts representative FLAIR axial slices in age- and sex-matched participants stratified by these three factors.

**Table 4:** Combined model of WMH load with most explanatory factors from Table 3, excluding diastolic BP and use of public transportation to reduce collinearities (*R*^2^ = 54%)

Variable	*β*	SE	*T*-statistic	*P*-value
Intercept	0.005	0.063	0.1	0.94
Age	0.495	0.069	7.2	<0.001
Sex	0.046	0.090	0.5	0.61
eTIV	0.291	0.084	3.5	<0.001
Urbanicity	**0.169**	**0.068**	**2.5**	**0.01**
BMI	**0.262**	**0.068**	**3.9**	**<0.001**
Store trip frequency	**−0.154**	**0.067**	**−2.3**	**0.02**
Daily chore hours	**−0.162**	**0.070**	**−2.3**	**0.02**
Systolic BP	**0.278**	**0.064**	**4.3**	**<0.001**

Rows in bold are those that were significant after multiple comparisons correction, with FDR *P*-value <0.05.

Comparing the 93 urban and 33 rural participants in the neuroimaging cohort across the other 24 measures, we found that urban participants were more likely to be exposed to outdoor air pollution [PM2.5; odds ratio (OR) 5.61, FDR *P* < 0.001] but less likely to be exposed to unclean household fuel (OR 0.38, FDR *P* < 0.001; Supplementary Table 1). There were no other differences after multiple comparisons correction. We also tested for recruitment biases in the neuroimaging cohort, comparing both the urban and rural participants from the neuroimaging cohort to their counterparts in the overall LASI-DAD study (Supplementary Tables 2 and 3). We found that urban neuroimaging participants were more likely to work frequently (OR 1.25, FDR *P* < 0.001) and less likely to be exposed to outdoor air pollution (PM 2.5; OR 0.99, FDR *P* < 0.02) than urban participants from the overall study. Rural neuroimaging participants differed from other rural LASI-DAD participants in a number of ways: they were less likely to be exposed to outdoor air pollution (OR 0.95, FDR *P* < 0.001), more likely to watch more hours of TV per day (OR 1.54, FDR *P* = 0.009), less likely to use unclean cooking fuel (OR 0.27, FDR *P* = 0.009), more likely to make frequent store trips (OR 1.34, FDR *P* = 0.03), more likely to have higher BMI (OR 1.1, FDR *P* = 0.03), more likely to spend time doing household chores (OR 1.37, FDR *P* = 0.03), more likely to use public transportation (OR 3.79, FDR *P* = 0.03) and less likely to be frequently depressed (OR 0.52, FDR *P* = 0.03).

Finally, we repeated our analysis to account for reverse causality by additionally controlling for concurrent neurodegeneration and cognitive impairment. To do this, we built a baseline model that included hippocampal volume, cortical volume and HMSE score as additional covariates (Supplementary Table 4). This model explained 36% of the variance in WMH load. Supplementary Table 5 shows associations for the same 25 measures as before. We identified the same seven factors, which, in general, were only slightly attenuated due to these additional covariates: systolic and diastolic BP explained 8.1 and 3.9% of the variance, respectively (*t* = 4.1, FDR *P* < 0.001; *t* = 2.8, FDR *P* = 0.03, respectively), BMI explained an additional 9.0% (*t* = 4.5, FDR *P* < 0.001), daily chore hours explained an additional 3.9% (*t* = −2.8, FDR *P* = 0.03), urbanicity explained an additional 5.3% (*t* = 3.3, FDR *P* = 0.01), store trip frequency explained an additional 4.4% (*t* = −2.9, FDR *P* = 0.02) and public transportation usage explained an additional 3.4% (*t* = −2.4, FDR *P* = 0.03). In this case, two additional factors reached significance: literacy, which explained an additional 3.6% (*t* = 2.7, FDR *P* = 0.03), and air pollution exposure, which explained an additional 3.4% (*t* = 2.6, FDR *P* = 0.03).

## Discussion

This study investigated associations between WMH load and various socioeconomic, health, lifestyle and environmental measures of older adults living in India. Using a neuroimaging sub-study data from a large, population-representative study of ageing and dementia in India, we identified several factors that help to explain WMH load. We found that increased systolic BP, increased BMI and living in an urban setting were all associated with increased WMH load. In contrast, increased frequency of store or market trips and increased daily hours spent doing chores (household chores, maintenance or gardening) were both associated with decreased WMH load. A model with these five factors combined explained substantially more variance than a baseline model containing age, sex and head size (54 versus 27%).

A number of studies have found associations between vascular risk factors and WMHs. Hypertension, defined as BP above 130 (systolic) and 90 mm Hg (diastolic), in both mid-life and late life has been associated with increased concurrent and prospective WMH volume.^2,26–33^ Recent work using the UK Biobank found that systolic BP is more strongly associated with WMH load after age 50 while diastolic BP is more strongly associated with WMH load before age 50.^26^ Thus, our finding that systolic BP explained more variance in WMH load than diastolic BP (Table 3) may be due to the LASI-DAD study’s focus on older adults (Table 1). In the light of our findings and those of previous studies, controlling both systolic and diastolic BP in the Indian population, with its high rate of hypertension, may be especially important for reducing WMH load.^34^

Several previous studies have found an association between obesity and WMH load.^35–38^ Though obesity is a risk factor for hypertension, Lampe *et al.*^38^ found that obesity affects WMH load independently of hypertension through an increase in proinflammatory cytokines such as interleukin-6. Our study found that both BMI and systolic BP were associated with WMH load (Table 4), supporting the notion that these two factors may affect WMHs through different pathological mechanisms.

Physical activity levels and motor function have also been found to be associated with WMH volume.^39,40^ While we found no direct association between the reported frequency of exercise and WMH load, this may be due to the fact that only 15% of neuroimaging sub-study participants were reported to engage in exercise on a regular basis (Table 1). Similarly, over half of the participants (54%) were reported to never go for walks. A better proxy for physical activity and motor function appears to be daily hours spent doing household chores, maintenance or gardening: 79% of sub-study participants were reported as spending at least half an hour a day engaged in this type of physical activity. Similarly, 70% of sub-study participants were reported as making a store or market trip at least once a week. We found that long hours spent doing chores and more frequent trips to the store were both associated with less WMH load, even after accounting for systolic BP and BMI (Table 4). There are several potential explanations for these associations. Prospective studies of older adults have shown that cardiovascular fitness is associated with both increased grey and white matter volume^41^ and white matter integrity,^42^ suggesting a neuroprotective effect of physical activity.^43^ Alternatively, the damaged white matter may lead to reduced physical activity via impaired mobility, gait or balance dysfunction as well as reduced cognitive ability, suggesting reverse causality.^44,45^ Accounting for such reverse causality by additionally controlling for concurrent neurodegeneration and cognitive impairment did not substantially impact our findings. Therefore, while our study does not establish causality, our findings support the idea that increased physical activity reduces WMH load.

Our study is one of the first to investigate the effects of urbanicity on brain structure. We found that those living in an urban setting have a higher WMH load (Table 4). In trying to understand this effect, we compared urban and rural neuroimaging sub-study participants, finding a difference in exposure to both indoor and outdoor air pollution between groups (Supplementary Table 1). However, these factors had no direct associations with WMH load (Table 3). There are likely other health, lifestyle or environmental drivers of the urbanicity effect. We found a number of differences between rural sub-study participants and other rural participants from the overall LASI-DAD study, which is designed to represent the population of India. Generally, rural sub-study participants appear to be healthier and wealthier than other rural Indians, experiencing less exposure to indoor and outdoor air pollution, greater use of public transportation and more frequent trips to the store, lower frequency of depression, greater hours of TV watching and better education (Supplementary Table 3). This may be due to the recruitment of rural participants from within a 4 h driving radius of the neuroimaging sites; those living in very remote rural areas were not sampled. Our study highlights the difficulties in recruiting a representative cohort of rural participants to urban neuroimaging centres.

One of the strengths of our study is the broad set of dementia-related risk factors that we were able to explore thanks to the LASI-DAD study’s richly characterized cohort. This meant that we could go beyond well-established factors such as hypertension to explore associations with both self- and informant-reported measures of health and lifestyle as well as the socioeconomic and environmental measures. In doing so, we found a set of factors which combine to explain substantially more variance in WMH load than a simple baseline model with only age, sex and head size. Importantly, we also showed that a number of potential health, lifestyle and environmental factors do not strongly influence WMH load (Table 3 and Supplementary Table 5). As a result, our study is one of the most comprehensive characterizations of cerebrovascular pathology risk factors. It is also one of the first to focus on India, a socioeconomically diverse country with a large and growing elderly population.

This study had a number of limitations. As a cross-sectional, association-based study, we could not directly establish a causal link between the risk factors we identified and WMH load. While we controlled for concurrent neurodegeneration and cognitive impairment, showing that these did not have a substantial effect on our findings, we cannot rule out reverse causation, especially given that 39% of the neuroimaging cohort were at high risk of cognitive impairment (Table 1). In other words, there is a chance that cerebrovascular pathology may have led to changes in the health and lifestyle measures we identified rather than the other way around, as we suggest. A prospective, longitudinal study is needed to understand these measures’ effects over time to further validate these measures as risk factors. Another important limitation is that our study focuses on older adults whereas some of the factors identified by the Lancet Commission report, such as hypertension, traumatic brain injury (not tested in this study), alcohol consumption, obesity and lack of education may adversely affect the brain beginning in mid-life.^17,26^ Finally, we were limited by a recruitment of rural participants for the neuroimaging sub-study that did not represent the broader population of rural-living Indians, which we demonstrated by comparing rural sub-study participants to other rural LASI-DAD participants.

In summary, we have identified three key factors that contribute to WMH load in older Indians. Controlling high systolic BP, maintaining a healthy BMI and maintaining high levels of physical activity all contribute to reducing the severity of cerebrovascular pathology and may, in turn, help to prevent or delay subsequent cognitive decline. Our findings contribute to a growing body of work aimed towards understanding the modifiable risk factors that contribute to cerebrovascular pathology and neurodegeneration in older individuals. Our work expands upon this previous work in an under-represented cohort of participants from an LMIC, thereby enhancing the generalizability of future public health recommendations aimed at reducing the incidence of preventable dementia.

## Supplementary Material

fcad008_Supplementary_Data

## Data Availability

The demographic, socioeconomic, health, lifestyle and environmental data are from the LASI-DAD Wave 1 release and are available on request from the Gateway to Global Aging Website at https://g2aging.org/. Neuroimaging data from Wave 1 are available through the Image and Data Archive online database hosted by the Laboratory of Neuro Imaging at the University of Southern California. All available MRI modalities are available for download in digital imaging and communications in medicine (DICOM) or neuroimaging informatics technology initiative (NIFTI) file formats. Currently the LASI-DAD neuroimaging data are hosted under a restricted-sharing policy: permission can be requested by submitting a data use application at https://adni.loni.usc.edu/.

## Abbreviations

AD =: Alzheimer's disease
BHFDR =: Benjamini and Hochberg false discovery rate
BMI =: body mass index
BP =: blood pressure
DICOM =: digital imaging and communications in medicine
eTIV =: estimated total intracranial volume
FDR =: false discovery rate
FLAIR =: fluid-attenuated inversion recovery
FOV =: field-of-view
GRAPPA =: GeneRalized Autocalibrating Partial Parallel Acquisition
HMSE =: Hindi mental state exam
LMIC =: low- and middle-income countries
LPA =: lesion prediction algorithm
MPRAGE =: magnetization prepared rapid acquisition gradient echo sequence
NIFTI =: neuroimaging informatics technology initiative
OR =: odds ratio
PM2.5 =: fine inhalable particle matter with diameter 2.5 μm or smaller
SPM =: statistical parametric mapping
TE =: echo time
TR =: repitition time
WMHs =: white matter hyperintensities
