# Supplementary material for: Investigating the factors that explain white matter hyperintensity load in older Indians
Source: Brain Commun. 2023 Jan 18;5(1):fcad008. doi: 10.1093/braincomms/fcad008 (PMC9891346; doi:10.1093/braincomms/fcad008)
Supplement: fcad008_Supplementary_Data [file fcad008_supplementary_data.docx]

**Supplementary Table 1** Comparison of differences between 93 urban and 33 rural participants in the neuroimaging cohort (N=126).

| Variable | Odds Ratio | | P value | FDR P value |
| --- | --- | --- | --- | --- |
| Air Pollution Exposure (PM2.5) | | **5.61** | **<0.001** | **<0.001** |
| Uses Unclean Household Fuel | | **0.38** | **<0.001** | **<0.001** |
| Daily Chores | | 0.58 | 0.02 | 0.13 |
| Hearing Test | | 0.62 | 0.03 | 0.16 |
| Uses Unclean Cooking Fuel | | 0.67 | 0.03 | 0.16 |
| Literate | | 1.51 | 0.06 | 0.23 |
| Diastolic BP | | 1.44 | 0.11 | 0.34 |
| Systolic BP | | 1.45 | 0.10 | 0.34 |
| Education Years | | 1.28 | 0.26 | 0.58 |
| Exercise Frequency | | 1.51 | 0.22 | 0.58 |
| Napping Frequency | | 1.25 | 0.29 | 0.58 |
| Uses Public Transport | | 0.78 | 0.30 | 0.58 |
| Work Frequency | | 1.24 | 0.32 | 0.58 |
| Loneliness Frequency | | 0.84 | 0.39 | 0.67 |
| Prepares Hot Meal | | 0.83 | 0.48 | 0.77 |
| Daily Computer Use | | 1.19 | 0.57 | 0.82 |
| Depressed Frequency | | 1.12 | 0.61 | 0.82 |
| Daily TV Hours | | 1.12 | 0.61 | 0.82 |
| BMI | | 1.09 | 0.69 | 0.83 |
| Walking Frequency | | 0.91 | 0.66 | 0.83 |
| Reading Frequency | | 1.07 | 0.76 | 0.87 |
| Store Trips Frequency | | 0.97 | 0.87 | 0.93 |
| Smoking Frequency | | 0.97 | 0.89 | 0.93 |

Logistic regression models were used to find how each factor differentiates urbanicity status in the neuroimaging cohort. Each variable was used separately to predict urbanicity status (urban coded as one, rural as zero), controlling for age and sex in each case. Variables in bold are those that were significant after multiple comparisons correction, with FDR P value < 0.05.

**Supplementary Table 2** Comparison of factors differentiating urban participants in the neuroimaging cohort (N=93) from the overall LASI-DAD cohort (N=1463).

| Variable | Odds Ratio | | P value | FDR P value |
| --- | --- | --- | --- | --- |
| Work Frequency | | **1.25** | **<0.001** | **<0.001** |
| Air Pollution Exposure (PM2.5) | | **0.99** | **0.002** | **0.02** |
| Depressed Frequency | | 0.70 | 0.010 | 0.08 |
| Store Trips Frequency | | 1.14 | 0.03 | 0.16 |
| Uses Public Transport | | 1.80 | 0.03 | 0.16 |
| Daily Chores | | 1.14 | 0.07 | 0.19 |
| Daily Reading | | 0.79 | 0.05 | 0.19 |
| Daily TV Watching | | 1.17 | 0.06 | 0.19 |
| Diastolic BP | | 1.02 | 0.07 | 0.19 |
| Alcohol Frequency | | 1.31 | 0.10 | 0.23 |
| Literate | | 1.44 | 0.12 | 0.25 |
| Daily Computer Use | | 0.71 | 0.16 | 0.29 |
| Loneliness Frequency | | 0.85 | 0.23 | 0.39 |
| Daily Napping | | 0.88 | 0.12 | 0.25 |
| Prepares Hot Meal | | 0.79 | 0.35 | 0.57 |
| Exercise Frequency | | 0.95 | 0.50 | 0.74 |
| BMI | | 0.99 | 0.74 | 0.77 |
| Systolic BP | | 1.00 | 0.52 | 0.74 |
| Uses Unclean Cooking Fuel | | 0.88 | 0.72 | 0.77 |
| Education Years | | 0.99 | 0.67 | 0.77 |
| Hearing Test | | 0.97 | 0.62 | 0.77 |
| Uses Unclean Household Fuel | | 0.99 | 0.98 | 0.98 |
| Walking Frequency | | 0.98 | 0.73 | 0.77 |
| Smoking Frequency | | 1.01 | 0.61 | 0.77 |

Logistic regression models of how each listed factor differentiates urban neuroimaging participants versus urban LASI-DAD participants. Each variable was used separately to predict membership in the urban neuroimaging cohort (urban neuroimaging cohort participation coded as one, all other urban LASI-DAD participants coded as zero), controlling for age and sex in each case. Variables in bold are those that were significant after multiple comparisons correction, with FDR P value < 0.05.

**Supplementary Table 3** Comparison of factors differentiating rural participants in the neuroimaging cohort (N=33) from the overall LASI-DAD cohort (N=2506).

| Variable | Odds Ratio | | P value | FDR P value |
| --- | --- | --- | --- | --- |
| Air Pollution Exposure (PM2.5) | | **0.95** | **<0.001** | **<0.001** |
| Daily TV Watching | | **1.54** | **0.001** | **0.009** |
| Uses Unclean Cooking Fuel | | **0.27** | **<0.001** | **0.009** |
| Store Trips Frequency | | **1.34** | **0.004** | **0.03** |
| BMI | | **1.10** | **0.01** | **0.03** |
| Daily Chores | | **1.37** | **0.01** | **0.03** |
| Uses Public Transport | | **3.79** | **0.01** | **0.03** |
| Depressed Frequency | | **0.52** | **0.01** | **0.03** |
| Education Years | | 1.10 | 0.02 | 0.05 |
| Systolic BP | | 0.99 | 0.11 | 0.27 |
| Daily Napping | | 0.83 | 0.13 | 0.28 |
| Work Frequency | | 1.14 | 0.14 | 0.28 |
| Literate | | 1.65 | 0.19 | 0.36 |
| Uses Unclean Household Fuel | | 0.67 | 0.30 | 0.52 |
| Exercise Frequency | | 0.83 | 0.42 | 0.65 |
| Daily Reading | | 1.16 | 0.43 | 0.65 |
| Diastolic BP | | 0.99 | 0.49 | 0.66 |
| Prepares Hot Meal | | 1.33 | 0.50 | 0.66 |
| Loneliness Frequency | | 0.89 | 0.55 | 0.69 |
| Hearing Test | | 1.04 | 0.59 | 0.71 |
| Walking Frequency | | 0.97 | 0.73 | 0.83 |
| Alcohol Frequency | | 0.00 | 1.00 | 1.00 |
| Daily Computer Use | | 0.99 | 0.99 | 1.00 |
| Smoking Frequency | | 1.00 | 0.96 | 1.00 |

Logistic regression models of how each listed factor differentiates rural neuroimaging participants versus rural LASI-DAD participants. Each variable was used separately to predict membership in the rural neuroimaging cohort (rural neuroimaging cohort participation coded as one, all other rural LASI-DAD participants coded as zero), controlling for age and sex in each case. Variables in bold are those that were significant after multiple comparisons correction, with FDR P value < 0.05.

**Supplementary Table 4** Baseline model of WMH severity with additional HMSE, hippocampal volume and cortical volume covariates (R^2^ = 36%).

| Variable | $\boldsymbol{\beta}$ | SE | T statistic | P value |
| --- | --- | --- | --- | --- |
| Intercept | 0.000 | 0.073 | 0.0 | 1.00 |
| Age | 0.275 | 0.087 | 3.2 | 0.002 |
| Sex | 0.005 | 0.097 | 0.1 | 0.96 |
| eTIV | 0.575 | 0.123 | 4.7 | <0.001 |
| HMSE | 0.058 | 0.083 | 0.7 | 0.48 |
| Hippocampal Volume | -0.270 | 0.110 | -2.4 | 0.02 |
| Cortical Volume | -0.218 | 0.150 | -1.5 | 0.15 |

**Supplementary Table 5** Associations between socioeconomic, health, lifestyle and environmental measures and WMH load (with additional covariates in baseline model).

| Variable | Additional R^2^ (%) | | T statistic | P value | FDR P value |
| --- | --- | --- | --- | --- | --- |
| BMI | | **9.0** | **4.5** | **<0.001** | **<0.001** |
| Systolic BP | | **8.1** | **4.1** | **<0.001** | **<0.001** |
| Urbanicity | | **5.3** | **3.3** | **0.001** | **0.01** |
| Store Frequency | | **4.4** | **-2.9** | **0.004** | **0.02** |
| Diastolic BP | | **3.9** | **2.8** | **0.006** | **0.03** |
| Daily Chore Hours | | **3.9** | **-2.8** | **0.006** | **0.03** |
| Literate | | **3.6** | **2.7** | **0.009** | **0.03** |
| Uses Public Transport | | **3.4** | **-2.6** | **0.01** | **0.03** |
| Air Pollution Exposure (PM2.5) | | **3.4** | **2.6** | **0.01** | **0.03** |
| Work Frequency | | 2.9 | -2.4 | 0.02 | 0.05 |
| Depressed Frequency | | 2.2 | 2.0 | 0.04 | 0.10 |
| Prepares Hot Meals | | 1.4 | -1.7 | 0.10 | 0.21 |
| Exercise Frequency | | 1.0 | 1.3 | 0.19 | 0.36 |
| Daily Napping Hours | | 0.7 | 1.1 | 0.27 | 0.48 |
| Uses Unclean Household Fuel | | 0.5 | -0.9 | 0.35 | 0.59 |
| Daily TV Hours | | 0.4 | 0.9 | 0.39 | 0.62 |
| Daily Reading Hours | | 0.3 | 0.7 | 0.49 | 0.66 |
| Daily Computer Hours | | 0.2 | 0.7 | 0.50 | 0.66 |
| Education Years | | 0.1 | 0.4 | 0.71 | 0.80 |
| Walking Frequency | | 0.1 | 0.4 | 0.68 | 0.80 |
| Uses Unclean Cooking Fuel | | 0.1 | -0.5 | 0.63 | 0.78 |
| Loneliness Frequency | | 0.0 | 0.2 | 0.85 | 0.88 |
| Smoking Frequency | | -0.3 | -0.7 | 0.47 | 0.66 |
| Alcohol Frequency | | -0.6 | -0.1 | 0.89 | 0.89 |
| Hearing Test | | -1.0 | -0.3 | 0.74 | 0.80 |

Each variable was used separately to predict WMH load, controlling for age, sex, eTIV, HMSE, hippocampal volume, and cortical volume in each case. Additional explained variance (R^2^) percentage is calculated as difference between resulting model’s R^2^ and baseline model’s (age, sex, eTIV, HMSE, hippocampal vol., cortical vol.) R^2^. Variables in bold are those that were significant after multiple comparisons correction, with FDR P value < 0.05.
